# Association between the cumulative dose of glucocorticoids before the development of pneumonia and death in patients receiving long-term glucocorticoids: a secondary analysis based on a Chinese cohort study

**DOI:** 10.3389/fmed.2023.1175855

**Published:** 2023-07-20

**Authors:** Hui-Jie Guo, Yi-Lu Ye, Rong Cao, Zhi-Hua Liu, Qun He

**Affiliations:** ^1^Guangdong Provincial Institute of Public Health, Guangdong Provincial Center for Disease Control and Prevention, Guangzhou, China; ^2^Department of Rehabilitation Medicine, Key Laboratory of Biological Targeting Diagnosis, The Fifth Affiliated Hospital of Guangzhou Medical University, Guangzhou, China; ^3^State Key Laboratory of Organ Failure Research, Guangdong Provincial Key Laboratory of Viral Hepatitis Research, Department of Infectious Diseases, Nanfang Hospital, Southern Medical University, Guangzhou, China

**Keywords:** glucocorticoids, immunocompromised patients, pneumonia, case fatality risks, cohort study

## Abstract

**Background:**

The present study aimed to evaluate the association between the cumulative dose of glucocorticoids (GCs) and case fatality in hospitalized patients who developed pneumonia while receiving glucocorticoid therapy.

**Methods:**

This retrospective cohort study included 625 patients receiving long-term GC treatment who were hospitalized with pneumonia (322 male and 303 female). Data were obtained from the Dryad Digital Repository and were used to perform secondary analysis. Multivariable Cox proportional hazard regression model and restricted cubic splines (RCS) were used to evaluate the association between the cumulative dose of GCs and case fatality. Sensitivity analyses and subgroup analyses were performed.

**Results:**

The 30-day and 90-day death rates were 22.9 and 26.2%, respectively. After adjusting for potential confounders, compared with those in the lowest quintile (≤ 1.5 g), the Cox proportional hazard regression model analysis showed that patients with different cumulative doses of GCs (1.5 to 2.95, 2.95 to 5, 5 to 11.5, and > 11.5 g) had lower risks for 30-day death, with respective hazard ratios of 0.86 (95% CI, 0.52 to 1.42), 0.81 (0.49 to 1.33), 0.29 (0.15 to 0.55), and 0.42 (0.22 to 0.79). The multivariable-adjusted RCS analysis suggested a statistically significant N-shaped association between the cumulative dose of GCs and 30-day death. A higher cumulative dose of GC tended to first lead to an increase in 30-day death within 1.8 g, then to a statistically significant decrease until around 8 g [HR for 1 g = 0.82 (0.69 to 0.97)], and again to an increase afterward. Similar results were found in the subgroup analyses and sensitivity analyses.

**Conclusion:**

N-shaped association between the cumulative dose of GCs and case fatality was observed in patients receiving long-term GC treatment who were hospitalized with pneumonia. Our findings may help physicians manage these patients.

## 1. Introduction

Glucocorticoids (GCs) are used extensively for the treatment of inflammatory and suppressing immune responses (1, 2). Prolonged GC treatment may result in severe immunosuppression and opportunistic infections such as pneumonia due to a decisive influence on the immune function of macrophages and granulocytes (3–9). Numerous reports of opportunistic pulmonary infections (mainly caused by *Pneumocystis jirovecii*, *Aspergillus*, and *Cytomegalovirus*) have been described in immunocompromised patients receiving GCs (4–6, 10–17). Death rates of up to 50% have been reported in patients with immune-related diseases treated with long-term GCs who develop pulmonary infections (4, 6, 10). Given the high case fatality, it is imperative to identify the prognostic factors in patients who develop pneumonia while receiving GCs as early as possible and to enhance disease management.

Although several studies have been done to examine prognostic factors for pneumonia in non-HIV-infected immunosuppressed patients, researches focusing specifically on GC-induced immunocompromised patients are still scarce (12, 18–24). The effect of different doses of GCs before pneumonia onset on case fatality of pneumonia has been examined by a few studies in patients treated with long-term GCs (4, 10, 25). Patients with High-dose GCs seem to have a higher case fatality risk (4, 10, 25, 26). A meta-analysis demonstrated that the combined use of GCs with other immunosuppressants before infection resulted in poorer prognoses in autoimmune inflammatory disease patients with pneumocystis pneumonia (PCP) (3). A small sample study of 33 pulmonary infiltrates patients found that compared with patients receiving short-term GC treatment or receiving non-GC treatment, patients receiving long-term GC treatment showed the lowest local and systemic pulmonary inflammatory responses which are linked to the outcome of pneumonia (6). These previous studies have focused on the effects of High-dose of GCs, long-term GC treatment, and combined use of GCs with other immunosuppressants on the prognosis of GC-induced immunocompromised patients with pneumonia. The variable of cumulative exposure dosage is a combined measure of the daily dose and duration of GC use, which facilitates comprehensive and comparable quantitative assessments of GC use. With the extensive use of GCs and the accumulation of patients receiving long-term GCs, assessing the effect of cumulative doses of GCs on case fatality after pulmonary infections is important for disease management. However, the association between the cumulative dose of GCs before infection and case fatality in GC-induced immunocompromised patients with pneumonia is still unclear. In this study, we evaluated the dose–response relationship between the cumulative dose of GCs and case fatality utilizing 625 GC-induced immunocompromised patients hospitalized with pneumonia between January 1, 2013 and December 31, 2017 at six secondary and tertiary academic hospitals in China.

## 2. Materials and methods

### 2.1. Study design and participants

The data used in this study were obtained from the Dryad Digital Repository website.^1^ This website makes the raw data of published papers freely reusable for secondary analysis. According to the Dryad Terms of Service, Dryad data package was cited in the current study (27). In the original report (25), Li et al. conducted a retrospective cohort study of patients with pneumonia at six secondary and tertiary academic hospitals in China. 716 patients with underlying immune-related diseases treated chronically with GCs who were hospitalized with pneumonia between 1 January 2013 and 31 December 2017 were recruited. We downloaded the raw data and performed a secondary analysis. No informed consent was required because the data were anonymized.

### 2.2. Data collection

We extracted variables from the previously mentioned database as follows: Demographic data, medical history, clinical symptoms, initial vital signs and lung examination findings, disease severity [indicated by intensive care unit (ICU) admission, use of invasive or non-invasive mechanical ventilation, pneumonia severity index (PSI) score and/or confusion, uremia, elevated respiratory rate, hypotension, and aged 65 years or older (CURB-65 score)], laboratory and microbiological data, treatment information before admission (including use of antibiotics, antiviral drugs, GCs, and other immunosuppressants), and survival status 30 and 90-days after admission. The exposure variable was the cumulative dose of GCs, defined as the cumulative dose of oral or intravenous GC treatment for underlying immune-related diseases before developing pneumonia. More specific details are presented in the original report (25).

### 2.3. Outcomes

The primary outcome was 30-day death after admission, and secondary outcomes included ICU admission, respiratory failure, mechanical ventilation, CURB-65 score greater than 1, PSI score, multi-drug resistance (MDR), and persistent lymphocytopenia.

### 2.4. Statistical analyses

We excluded 88 patients who had no available information on the cumulative dose of GC treatment. Three additional patients also were excluded because of unrealistic values. Continuous variables were expressed as mean ± SD, and categorical variables were expressed as numbers (%). Differences were tested using the $\chi^{2}$ test for categorical variables and the ANOVA for continuous variables.

The cumulative dose of GCs was divided into quintiles. Hazard ratios (HRs) and 95% confidence intervals (CIs) of those having a higher cumulative dose of GCs were estimated by Cox proportional hazard regression models with the lowest quintiles as the reference class. We developed three Cox proportional hazard regression models to evaluate the association between the cumulative dose of GCs and 30-day death. We did not adjust for any covariate in Model I and adjusted for age, sex, smoking status, and alcoholism in Model II. Model II plus high-dose steroids use, receiving other immunosuppressants, Pre-admission antibiotics treatment, Pre-admission antiviral drugs treatment, underlying immune-related diseases for chronic GC treatment, and microbial etiology of pneumonia constituted Model III, which was the fully adjusted model.

We also used restricted cubic splines (RCS) with five knots at the 5th, 27.5th, 50th, 72.5th, and 95th centiles to flexibly model the dose–response relationship between the cumulative dose of GCs and 30-day death in the fully adjusted model. We tested for potential non-linearity by using an ANOVA test comparing the model with only a linear term against the model with linear and cubic spline terms. If the relationship was nonlinear, we applied a segmented regression analysis to determine inflection points where the slope of the RCS curve changed suddenly. Then we built multivariable Cox proportional hazard regression models on either side of these points to investigate the association of the cumulative dose of GCs with 30-day death.

In the subgroup analyses, adjusted HRs and RCS smoothing plots of 30-day death associated with the cumulative dose were estimated and created, respectively, stratified by age (< 60 and ≥ 60 years), sex, smoking status, high-dose steroids use, receiving other immunosuppressants, connective tissue disease, interstitial lung disease, and microbial pathogens (*Cytomegalovirus, Pneumocystis*, and Gram-negative bacteria). The heterogeneity of effects across the subgroups was assessed by fitting models with and without interaction terms and comparing these by the likelihood ratio test. In addition, to examine the robustness of our findings, we performed a series of sensitivity analyses by conducting a propensity-score matching analysis and using 90-day death as the outcome variable. For the propensity-score matching, we matched patients by the aforementioned potential confounders. Matching was conducted with the use of a 1:2 matching protocol and with a propensity score in a range of ±0.02. Statistical analyses were performed using statistical packages R (version 4.1.1, The R Foundation; http://www.r-project.org). All the tests were 2-sided, and a *p* < 0.05 was considered statistically significant.

## 3. Results

### 3.1. Baseline characteristics

A total of 625 adult patients (322 male and 303 female) were enrolled in this study. The most common underlying immune-related diseases were connective tissue disease (53.1%) and interstitial lung disease (49.1%). The baseline characteristics of patients are summarized in Table 1. Patients with a higher cumulative dose of GCs were significantly more likely to be female predominant, more likely to have connective tissue disease, and had lower percentages of smoking (*p* < 0.05). Significant differences were found among groups in terms of the proportion of alcoholism, High-dose steroids use, receiving other immunosuppressants, Pre-admission antiviral drugs use, nephrotic syndrome or chronic glomerulonephritis, bronchial asthma or chronic obstructive pulmonary disease, lymphoma, solid organ transplant, and microbial pathogens, including *Cytomegalovirus*, *Pneumocystis*, *Aspergillus*, and Gram-negative bacteria (All *p* < 0.05). In addition, Supplementary Table S1 shows the distributions of symptoms, signs, and laboratory examinations.

**Table 1:** Baseline characteristics according to the cumulative dose of GCs in study participants.

Variables, *n* (%)	Total	Q1 (≤1.5 g)	Q2 (1.5–2.95 g)	Q3 (2.95–5 g)	Q4 (5–11.5 g)	Q5 (>11.5 g)	*p* value
30-day death	143(22.9)	37 (29.6)	37 (28.5)	36 (30.0)	15 (12.0)	18 (14.4)	<0.001
Sex, female	303 (48.5)	47 (37.6)	53 (40.8)	51 (42.5)	65 (52.0)	87 (69.6)	<0.001
Age							0.362
18–39	86 (13.8)	12 (9.6)	15 (11.5)	20 (16.7)	21 (16.8)	18 (14.4)	
40–49	84 (13.4)	14 (11.2)	11 (8.5)	18 (15.0)	18 (14.4)	23 (18.4)	
50–59	131 (21.0)	25 (20.0)	35 (26.9)	24 (20.0)	29 (23.2)	18 (14.4)	
60–69	193 (30.9)	43 (34.4)	46 (35.4)	33 (27.5)	33 (26.4)	38 (30.4)	
70–79	90 (14.4)	22 (17.6)	16 (12.3)	20 (16.7)	15 (12.0)	17 (13.6)	
80–99	41 (6.6)	9 (7.2)	7 (5.4)	5 (4.2)	9 (7.2)	11 (8.8)	
Smoking							0.003
Never	464 (74.2)	81 (64.8)	91 (70.0)	83 (69.2)	100 (80.0)	109 (87.2)	
Current	148 (23.7)	40 (32.0)	35 (26.9)	33 (27.5)	24 (19.2)	16 (12.8)	
Former	13 (2.1)	4 (3.2)	4 (3.1)	4 (3.3)	1 (0.8)	0 (0.0)	
Alcoholism	49 (7.8)	10 (8.0)	10 (7.7)	16 (13.3)	10 (8.0)	3 (2.4)	0.038
High-dose steroids^a^	237 (37.9)	47 (37.6)	62 (47.7)	71 (59.2)	42 (33.6)	15 (12.0)	<0.001
Other immunosuppressants^b^	237 (37.9)	59 (47.2)	58 (44.6)	60 (50.0)	38 (30.4)	22 (17.6)	<0.001
Pre-admission antibiotics	429 (68.6)	86 (68.8)	85 (65.4)	92 (76.7)	80 (64.0)	86 (68.8)	0.241
Pre-admission antiviral drugs	102 (16.3)	26 (20.8)	20 (15.4)	27 (22.5)	17 (13.6)	12 (9.6)	0.039
Diabetes mellitus	159 (25.4)	34 (27.2)	36 (27.7)	30 (25.0)	33 (26.4)	26 (20.8)	0.728
Tumor	37 (5.9)	5 (4.0)	9 (6.9)	6 (5.0)	12 (9.6)	5 (4.0)	0.276
Connective tissue disease	332 (53.1)	51 (40.8)	53 (40.8)	57 (47.5)	73 (58.4)	98 (78.4)	<0.001
Interstitial lung disease	307 (49.1)	61 (48.8)	64 (49.2)	55 (45.8)	67 (53.6)	60 (48.0)	0.81
Nephrotic syndrome or chronic glomerulonephritis	79 (12.6)	9 (7.2)	19 (14.6)	22 (18.3)	19 (15.2)	10 (8.0)	0.033
Idiopathic interstitial pneumonia	63 (10.1)	12 (9.6)	15 (11.5)	16 (13.3)	15 (12.0)	5 (4.0)	0.119
Bronchial asthma or chronic obstructive pulmonary disease	15 (2.4)	9 (7.2)	4 (3.1)	0 (0.0)	2 (1.6)	0 (0.0)	0.001
Lymphoma	13 (2.1)	0 (0.0)	7 (5.4)	4 (3.3)	2 (1.6)	0 (0.0)	0.01
Bone marrow or hematopoietic stem cell transplant	5 (0.8)	3 (2.4)	0 (0.0)	0 (0.0)	2 (1.6)	0 (0.0)	0.089
Solid organ transplant	60 (9.6)	27 (21.6)	18 (13.8)	6 (5.0)	2 (1.6)	7 (5.6)	<0.001
Radiation pneumonitis	6 (1.0)	0 (0.0)	1 (0.8)	1 (0.8)	4 (3.2)	0 (0.0)	0.059
Pathogens							
Viruses							
Cytomegalovirus	202 (32.3)	27 (21.6)	60 (46.2)	47 (39.2)	39 (31.2)	29 (23.2)	<0.001
Influenza virus	76 (12.2)	23 (18.4)	12 (9.2)	17 (14.2)	11 (8.8)	13 (10.4)	0.102
Respiratory syncytial virus	63 (10.1)	18 (14.4)	14 (10.8)	8 (6.7)	12 (9.6)	11 (8.8)	0.349
Funguses							
Pneumocystis	138 (22.1)	22 (17.6)	35 (26.9)	39 (32.5)	27 (21.6)	15 (12.0)	0.001
Aspergillus	89 (14.2)	28 (22.4)	24 (18.5)	13 (10.8)	9 (7.2)	15 (12.0)	0.004
Bacteria							
Gram-negative	173 (27.7)	47 (37.6)	28 (21.5)	37 (30.8)	31 (24.8)	30 (24.0)	0.032
Gram-positive	58 (9.3)	16 (12.8)	12 (9.2)	11 (9.2)	7 (5.6)	12 (9.6)	0.424

a High-dose steroid use was defined as equal to or greater than 30 mg/day of prednisolone or an equivalent GC within 30-days before admission.

b Other immunosuppressants including methotrexate, cyclosporine, cyclophosphamide, tacrolimus, sirolimus, and azathioprine. Q, quintile.

### 3.2. Association between cumulative dose of GCs and case fatality

During up to 30 and 90-days of follow-up, we identified 143 (22.9%) and 164 (26.2%) deaths, respectively. Table 2 shows the results of the Cox proportional hazard regression models. The multivariable-adjusted model (Model III) revealed that compared with those in the lowest quintile (≤ 1.5 g), patients in different quintiles of the cumulative dose of GCs (1.5 to 2.95, 2.95 to 5, 5 to 11.5, and > 11.5 g) had lower risks for 30-day death, with respective hazard ratios of 0.86 (95% CI, 0.52 to 1.42), 0.81 (0.49 to 1.33), 0.29 (0.15 to 0.55), and 0.42(0.22 to 0.79).

**Table 2:** Hazard ratios (95% CIs) for the association between the cumulative dose of GC use and 30-day death by Cox regression models.

	Crude		Adjust model I^a^		Adjust model II^b^	
	HR (95% CI)	*p* value	HR (95% CI)	*p* value	HR (95% CI)	*p* value
Q1	1	–	1	–	1	–
Q2	0.99 (0.63, 1.56)	0.958	0.99 (0.62, 1.56)	0.949	0.86 (0.52, 1.42)	0.559
Q3	1.03 (0.65, 1.62)	0.911	1.04 (0.65, 1.65)	0.882	0.81 (0.49, 1.33)	0.4
Q4	0.37 (0.20, 0.67)	0.001	0.36 (0.20, 0.66)	0.001	0.29 (0.15, 0.55)	<0.001
Q5	0.44 (0.25, 0.78)	0.005	0.44 (0.25, 0.79)	0.006	0.42 (0.22, 0.79)	0.007

a Adjusted for age, sex, smoking status, and alcoholism.

b Adjusted for age, sex, smoking status, drinking status, alcoholism, high-dose steroids use, receiving other immunosuppressants, Pre-admission antibiotics, Pre-admission antiviral drugs, diabetes mellitus, tumor, connective tissue disease, interstitial lung disease, nephrotic syndrome or chronic glomerulonephritis, idiopathic interstitial pneumonia, bronchial asthma or chronic obstructive pulmonary disease, lymphoma, bone marrow or hematopoietic stem cell transplant, solid organ transplant, radiation pneumonitis, microbial etiology of pneumonia (including cytomegalovirus, influenza virus, respiratory syncytial virus, pneumocystis, aspergillus, Gram-negative and Gram-positive bacteria). Q, quintile.

**Figure 1:**
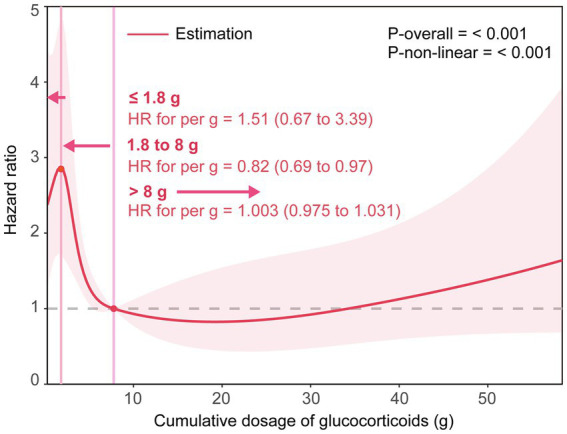
The smoothing curve of the dose–response relationship between cumulative dose of GCs and 30-day death. Adjusted for age, sex, smoking status, drinking status, alcoholism, high-dose steroids use, receiving other immunosuppressants, Pre-admission antibiotics, Pre-admission antiviral drugs, diabetes mellitus, tumor, connective tissue disease, interstitial lung disease, nephrotic syndrome or chronic glomerulonephritis, idiopathic interstitial pneumonia, bronchial asthma or chronic obstructive pulmonary disease, lymphoma, bone marrow or hematopoietic stem cell transplant, solid organ transplant, radiation pneumonitis, microbial etiology of pneumonia (including cytomegalovirus, influenza virus, respiratory syncytial virus, pneumocystis, aspergillus, Gram-negative and Gram-positive bacteria). The solid line represents the HRs, and the shaded areas represent the 95% confidence intervals for the spline model.

The multivariable-adjusted RCS analysis suggested a statistically significant N-shaped association of the cumulative dose of GCs with 30-day death (*p* for non-linearity ≤0.001). A higher cumulative dose of GC tended to first lead to an increase in 30-day death within 1.8 g [HR for 1 g = 1.51 (0.67 to 3.39)], then to a statistically significant decrease until around 8 g [HR for 1 g = 0.82 (0.69 to 0.97)], and again to an increase afterward [HR for 1 g = 1.003(0.975 to 1.031)] (Figure 1).

### 3.3. Subgroup and sensitivity analyses

Table 3 and Supplementary Figure S1 present results from subgroup analyses based on Cox models and RCS, respectively. The results of subgroup analyses based on Cox models overall did not suggest significant effect modification by stratification variables for 30-day death, and tests for interactions were not statistically significant (*p* interactions > 0.05), except for CMV infection status (*p* interaction = 0.036). Furthermore, regardless of the subgroup, the RCS smoothing plots suggested an N-shaped association of the cumulative dose of GCs with 30-day death.

**Table 3:** Results of the subgroup analyses based on Cox models.

		Events/ Total	Q2 HR (95% CI)	Q3 HR (95% CI)	Q4 HR (95% CI)	Q5 HR (95% CI)	*p* for interaction
Age, year							0.840
	<60	63/301	1.02 (0.45, 2.30)	1.16 (0.53, 2.56)	0.44 (0.16, 1.16)	0.49(0.18,1.33)	
	≥60	80/324	0.87 (0.45, 1.66)	0.60 (0.31, 1.18)	0.23 (0.09, 0.56)	0.35 (0.15, 0.80)	
Gender							0.746
	Male	75/322	0.76 (0.38, 1.52)	0.73 (0.38, 1.40)	0.23 (0.09, 0.59)	0.17 (0.05, 0.57)	
	Female	68/303	1.05 (0.47, 2.34)	0.86 (0.38, 1.96)	0.37 (0.14, 0.95)	0.62 (0.26, 1.47)	
Smoking							0.325
	No	103/464	0.98 (0.54, 1.77)	0.69 (0.36, 1.33)	0.33 (0.16, 0.68)	0.46 (0.23, 0.92)	
	Yes	40/161	0.43 (0.14, 1.35)	1.03 (0.39, 2.74)	0.14 (0.03, 0.80)	0.21 (0.03, 1.70)	
High-dose steroids							0.870
	No	69/388	0.69 (0.30, 1.58)	1.23 (0.54, 2.80)	0.26 (0.11, 0.66)	0.34 (0.15, 0.78)	
	Yes	74/237	0.97 (0.49, 1.95)	0.78 (0.38, 1.59)	0.33 (0.12, 0.95)	0.42 (0.12, 1.52)	
Other immunosuppressants							0.713
	No	88/388	0.99 (0.48, 2.02)	0.73 (0.36, 1.49)	0.23 (0.10, 0.53)	0.31 (0.14, 0.67)	
	Yes	55/237	1.11 (0.49, 2.55)	1.40 (0.66, 2.97)	0.46 (0.16, 1.35)	0.63 (0.17, 2.39)	
Connective tissue disease							0.709
	No	60/293	0.75 (0.34, 1.68)	0.82 (0.38, 1.79)	0.18 (0.06, 0.58)	0.74 (0.22, 2.47)	
	Yes	83/332	0.91 (0.46, 1.82)	0.82 (0.42, 1.61)	0.30 (0.13, 0.69)	0.30 (0.14, 0.66)	
Interstitial lung disease							0.898
	No	68/318	0.77 (0.34, 1.78)	0.71 (0.32, 1.58)	0.24 (0.08, 0.67)	0.58 (0.22, 1.49)	
	Yes	75/307	0.86 (0.43, 1.72)	0.79 (0.39, 1.59)	0.28 (0.11, 0.67)	0.30 (0.12, 0.75)	
**Pathogens**							
Cytomegalovirus							0.036
	No	89/423	1.42 (0.77, 2.63)	1.10 (0.58, 2.08)	0.41 (0.17, 0.96)	0.44 (0.20, 0.99)	
	Yes	54/202	0.31 (0.12, 0.82)	0.54 (0.21, 1.43)	0.17 (0.05, 0.55)	0.37 (0.11, 1.29)	
Pneumocystis							0.128
	No	101/487	0.96 (0.53, 1.77)	0.61 (0.33, 1.14)	0.32 (0.16, 0.66)	0.34 (0.16, 0.71)	
	Yes	42/138	0.88 (0.28, 2.74)	1.17 (0.43, 3.23)	0.14 (0.02, 0.89)	0.50 (0.10, 2.56)	
Gram-negative bacteriums							0.323
	No	95/452	0.44 (0.14, 1.45)	0.61 (0.25, 1.50)	0.27 (0.08, 0.89)	0.45 (0.14, 1.40)	
	Yes	48/173	1.17 (0.63, 2.17)	0.88 (0.46, 1.70)	0.28 (0.12, 0.64)	0.33 (0.14, 0.74)	

Adjusted for age, sex, smoking status, drinking status, high-dose steroids use, receiving other immunosuppressants, Pre-admission antibiotics, Pre-admission antiviral drugs, diabetes mellitus, tumor, connective tissue disease, interstitial lung disease, nephrotic syndrome or chronic glomerulonephritis, idiopathic interstitial pneumonia, bronchial asthma or chronic obstructive pulmonary disease, lymphoma, bone marrow or hematopoietic stem cell transplant, solid organ transplant, radiation pneumonitis, microbial etiology of pneumonia (including cytomegalovirus, influenza virus, respiratory syncytial virus, pneumocystis, aspergillus, Gram-negative and Gram-positive bacteria). Not adjusted for the stratification variable itself. Q, quintile.

Our findings remained robust in several sensitivity analyses. The propensity-score matching analysis showed consistent results, in which 196 patients without death were matched to 119 patients with 30-day death after correcting for potential confounders (Supplementary Tables S2, S3; Supplementary Figure S2). Furthermore, results were similar when we used 90-day death as the outcome variable (Supplementary Table S4; Supplementary Figure S3).

### 3.4. Association between the cumulative dose of GCs and secondary outcomes

**Figure 2:**
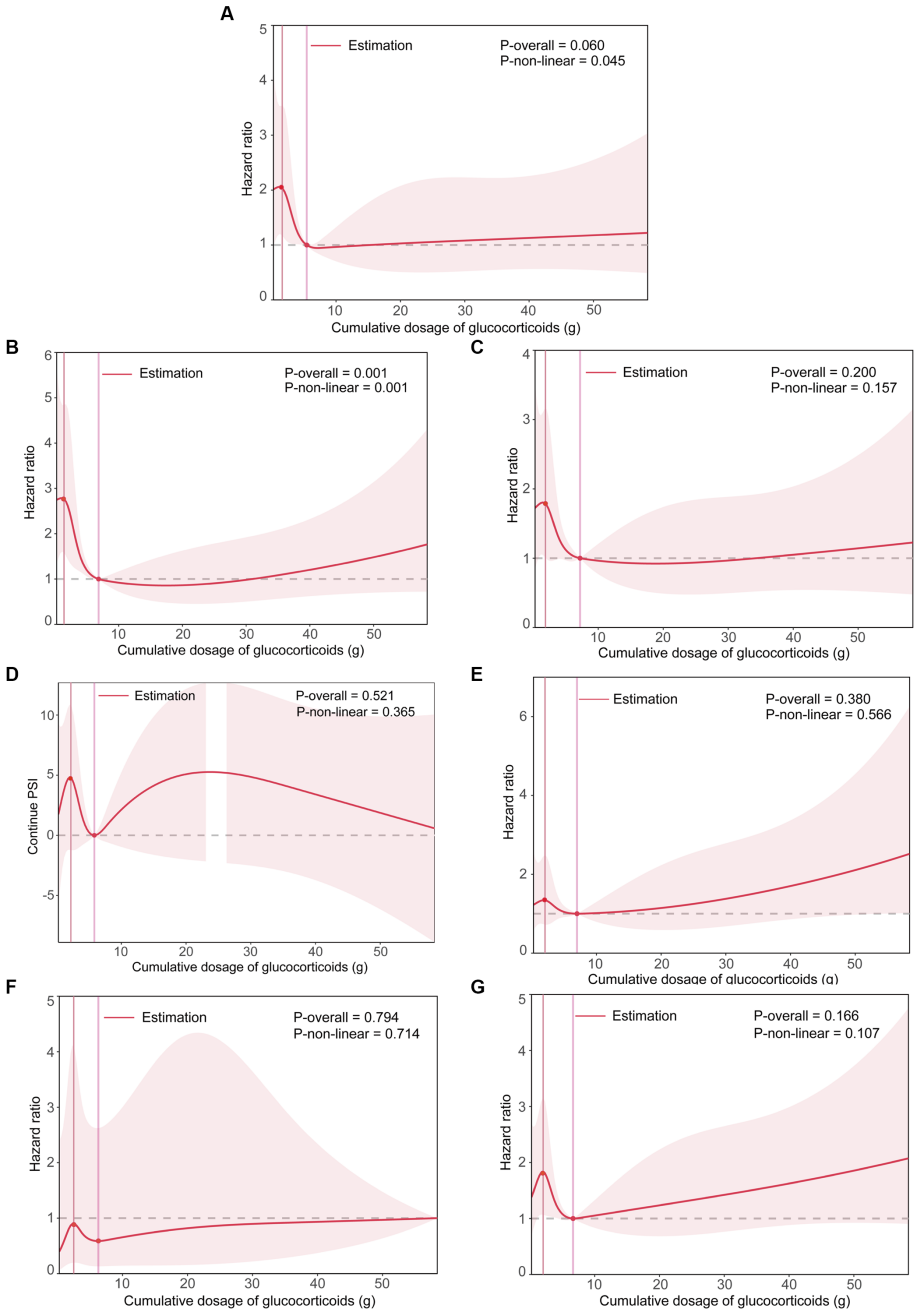
The smoothing curve of the dose–response relationship between cumulative dose of GCs and secondary outcomes. **(A)** Plot for ICU admission. **(B)** Plot for respiratory failure. **(C)** Plot for mechanical ventilation. **(D)** Plot for PSI score. **(E)** Plot for CURB-65 score > 1. **(F)** Plot for MDR. **(G)** Plot for persistent lymphocytopenia. Adjusted for age, sex, smoking status, drinking status, high-dose steroids use, receiving other immunosuppressants, Pre-admission antibiotics, Pre-admission antiviral drugs, diabetes mellitus, tumor, connective tissue disease, interstitial lung disease, nephrotic syndrome or chronic glomerulonephritis, idiopathic interstitial pneumonia, bronchial asthma or chronic obstructive pulmonary disease, lymphoma, bone marrow or hematopoietic stem cell transplant, solid organ transplant, radiation pneumonitis, microbial etiology of pneumonia (including cytomegalovirus, influenza virus, respiratory syncytial virus, pneumocystis, aspergillus, Gram-negative and Gram-positive bacteria). The solid line represents the HRs, and the shaded areas represent the 95% confidence intervals for the spline model. ICU, intensive care unit; PSI, pneumonia severity index; CURB-65, confusion, uremia, elevated respiratory rate, hypotension, and aged 65 years or older; MDR, multidrug-resistant.

Significant differences were found among different cumulative dose groups of GCs in terms of the proportions of ICU admission, respiratory failure, mechanical ventilation, and the PSI score (All *p* < 0.05) (Table 4). Although statistically significant for only ICU admission and respiratory failure risk (Both *p* for non-linearity <0.05), the RCS smoothing plots suggested similar N-shaped associations between the cumulative dose of GCs and each of the secondary outcomes (Figure 2).

**Table 4:** Comparisons of secondary outcomes among different cumulative dose groups of GCs.

	Q1	Q2	Q3	Q4	Q5	*p* value
ICU admission, *n* (%)	63 (50.4)	63 (48.5)	64 (53.3)	47 (37.6)	40 (32.0)	0.002
Respiratory failure, *n* (%)	78 (62.4)	77 (59.2)	76 (63.3)	50 (40.0)	47 (37.6)	<0.001
Mechanical ventilation, *n* (%)	54 (43.2)	56 (43.1)	54 (45.0)	43 (34.4)	32 (25.6)	0.007
PSI score [mean (SD)]	84.99 (30.87)	87.50 (34.10)	80.79 (30.71)	77.28 (32.18)	77.30 (30.96)	0.033
CURB-65 score > 1, *n* (%)	43 (34.4)	43 (33.1)	35 (29.2)	31 (24.8)	38 (30.4)	0.505
MDR, *n* (%)	28 (22.4)	19 (14.6)	21 (17.5)	18 (14.4)	18 (14.4)	0.362
Persistent lymphocytopenia, *n* (%)	59 (47.2)	61 (46.9)	52 (43.3)	46 (36.8)	55 (44.0)	0.463

Persistent lymphocytopenia was defined as a peripheral blood lymphocyte count lower than 1 × 10^9^/L for greater than 7-days.

ICU, intensive care unit; PSI, pneumonia severity index; CURB-65, confusion, uremia, elevated respiratory rate, hypotension, and aged 65 years or older; MDR, multidrug-resistant; Q, quintile.

## 4. Discussion

In the retrospective cohort study of patients receiving long-term GCs who were hospitalized with pneumonia, we examined the association between the cumulative dose of GCs before the development of pneumonia and case fatality. We found a significant N-shaped association between the cumulative dose of GCs and 30-day death. Our findings may help physicians manage these patients.

Opportunistic pulmonary infections are the most frequent complications in non-HIV-infected immunocompromised patients and portend high mortality (28). The mortality rate ranges from 30 to 40% in non-HIV-infected immunocompromised patients, versus 10 to 20% in HIV-infected patients (29, 30). The GC-induced immunocompromised patients account for a growing fraction of the non-HIV-infected immunocompromised population and have higher death rates of up to 45% (4–6, 10–17). In our study, the 30-day and 90-day case fatality rates were 22.9 and 26.2% in GC-induced immunocompromised patients with pneumonia, respectively.

GC-related adverse effects are the result of on-target glucocorticoid receptor-mediated effects in that they are amplified manifestations of the normal effects of endogenous cortisol (31). Generally, it is believed that transactivation is responsible to a greater extent than transrepression for adverse effects (31, 32). The risk of infection is increased during GC treatment due to immunosuppression mediated by transrepression and transactivation effects on both the innate and the acquired immune systems (31, 33). Long-term treatment with high-dose steroids is a significant risk factor for PCP in patients with solid organ transplants, hematologic malignancies, and rheumatic diseases (4, 34–36). Patients receiving an initial dose of prednisone ≥60 mg/day showed a significantly higher PCP incidence than those in <60 mg/day subgroups (4). Our study found that PCP was detected in 17.6, 26.9, 32.5%, 21.6, and 12.0% of patients in quintile groups of the cumulative dose of GCs (≤1.5, 1.5 to 2.95, 2.95 to 5, 5 to 11.5, and > 11.5 g) respectively. The detection rate of PCP increased and then decreased across increasing exposure groups.

To our knowledge, this is one of the rare studies to assess the association between the cumulative dose of GCs and case fatality in GC-induced immunocompromised patients with pneumonia and is the first to investigate the nonlinear relationship between them. Several studies have evaluated the effects of High-dose of GC, long-term GC treatment, and combined use of GC with other immunosuppressants on the prognosis of GC-induced immunocompromised patients with pneumonia (3, 4, 6, 25). One of these studies showed that the in-hospital mortality was 50%, and all those who died were on high-dose GC at the time of PCP diagnosis in GC-induced immunocompromised patients. The degree of harm associated with GC treatment is related to daily dose, total duration of treatment, and cumulative dose (37). For each 1 g increase in cumulative glucocorticoid dose, the risk for adverse events was increased by 3% (38). However, there are no reports on the dose–response relationship between the cumulative dose of GCs and case fatality in GC-induced immunocompromised patients with pneumonia. In the present study, we found a significant N-shaped association between the cumulative dose of GCs and 30-day death. That is, a higher cumulative dose of GC tended to first lead to an increase in 30-day death within 1.8 g [HR for 1 g = 1.51 (0.67 to 3.39)], then to a statistically significant decrease until around 8 g [HR for 1 g = 0.82 (0.69 to 0.97)], and again to an increase afterward [HR for 1 g = 1.003 (0.975 to 1.031)]. Interestingly, a long-term follow-up study included 779 rheumatoid arthritis patients suggested that compared to patients who were not receiving corticosteroids, the mortality risk first decreased and then increased across increasing exposure groups (Cumulative dose <9, 9–39.9, and ≥ 40 g groups), with respective hazard ratios of 0.59 (0.36 to 0.95), 1.12 (0.76 to 1.64), and 1.74 (1.25 to 2.44) for All-cause mortality, and hazard ratios of 0.70 (0.37 to 1.31), 1.08 (0.63 to1.88), and 2.05 (1.29 to 3.27) for cardiovascular mortality (39). These results were partially similar to the findings of our analyses for the case fatality rate of pneumonia. It should be noted that several differences exist between the long-term follow-up study and our study in study design. Firstly, the study population of the long-term follow-up study is whole patients with rheumatoid arthritis, but ours is a specific population, GC-induced immunocompromised patients hospitalized with pneumonia. Secondly, the outcome variables of the long-term follow-up study were all-cause mortality and cardiovascular mortality, which are markedly different from ours. Thirdly, the dose–response relationship in the long-term follow-up study was based on cumulative dose ranks, while ours was based on a continuous variable. We attempted to explore the underlying mechanisms of the N-shaped association between the cumulative dose of GCs and case fatality found in our study using available data. The indicators of pneumonia severity, including ICU admission, respiratory failure, mechanical ventilation, CURB-65 score greater than 1, PSI score, MDR, and persistent lymphocytopenia were analyzed as secondary outcomes. The RCS analyses suggested that although statistically significant for only ICU admission and respiratory failure indicators, all the indicators of pneumonia severity showed an N-shaped association with case fatality similar to the case fatality. The development of respiratory failure, the need for mechanical ventilation, and lymphocytopenia have been shown to be the strongest predictors of death in immunocompromised patients with pneumonia. These findings suggest a possible explanation for the N-shaped association between the cumulative dose of GCs and case fatality. That is, firstly, during the early stages of GC treatment initiation, the body is challenged by both the disease itself and the sudden stimulation of GC, and if pulmonary infections occur, the case fatality will increase with increasing cumulative doses of GCs. In the second stage, below a threshold (around 8 g), the pre-existing underlying immune-related disease and resulting physical damage are gradually improved with GC treatment, and the immune responses are suppressed, so the inflammatory response and severity of pneumonia are diminished and the outcome is better after pulmonary infections occur. In the third stage, above the threshold (around 8 g), GC tolerance and disease relapse might occur, and if pulmonary infections occur, the severity of pneumonia is substantial and the case fatality will increase with increasing cumulative doses of GCs (i.e., with increasing duration of GC treatment).

To ensure the reliability of our findings, we performed several subgroup and sensitivity analyses. Although some differences between subgroups were observed due to insufficient sample sizes after stratification, the results of subgroup analyses based on Cox models did not show statistically significant effect modification by stratification variables for case fatality. In addition, regardless of the subgroup, the smoothing spline plots suggested an N-shaped association between the cumulative dose of GCs with 30-day death. The sensitivity analyses further confirmed that our findings are robust.

The present study has several limitations. Firstly, this was a secondary analysis using publicly available data from a retrospective cohort study, which may result in some recall bias. Secondly, although our analyses controlled for many potential confounders, relevant confounders not captured by the publicly available dataset cannot be considered. Thirdly, we did not consider the potential influences of *Pneumocystis jirovecii* pneumonia prophylaxis on the findings of this study due to the constraint of the original data. Fourthly, our study was conducted among Chinese, so this association may need to be cautious when generalization among other ethnicities.

## 5. Conclusion

We found an N-shaped association between the cumulative dose of GCs and case fatality in GC-induced immunocompromised patients hospitalized with pneumonia. Our findings may help physicians manage these patients.

## Data availability statement

The datasets presented in this study can be found in online repositories. The names of the repository/repositories and accession number(s) can be found at: https://datadryad.org/resource/doi:10.5061/dryad.mkkwh70x2.

## Ethics statement

Ethical approval was not provided for this study on human participants because the data used for the analysis comes from the public database and the original author had obtained the ethical approval from the Ethics Committee of China-Japan Friendship Hospital. Written informed consent for participation was not required for this study in accordance with the national legislation and the institutional requirements.

## Author contributions

H-JG and Y-LY designed the study and wrote the first draft, and H-JG, Y-LY, RC, Z-HL, and QH revised it. QH and RC supervised the study. H-JG analyzed and interpreted the data. All authors contributed to the article and approved the submitted version.

## Funding

This study was funded by Science and Technology Program of Guangzhou (grant number: 202002020053).

## Conflict of interest

The authors declare that the research was conducted in the absence of any commercial or financial relationships that could be construed as a potential conflict of interest.

## Publisher’s note

All claims expressed in this article are solely those of the authors and do not necessarily represent those of their affiliated organizations, or those of the publisher, the editors and the reviewers. Any product that may be evaluated in this article, or claim that may be made by its manufacturer, is not guaranteed or endorsed by the publisher.

## Footnotes

^1^https://datadryad.org/resource/doi:10.5061/dryad.mkkwh70x2

## References

[1] Waljee AK Rogers MA Lin P Singal AG Stein JD Marks RM. Short term use of oral corticosteroids and related harms among adults in the United States: population based cohort study. BMJ. (2017) 357:j1415. doi: 10.1136/bmj.j1415, PMID: 28404617 PMC6284230

[2] Fardet L Petersen I Nazareth I. Prevalence of long-term oral glucocorticoid prescriptions in the UK over the past 20 years. Rheumatology (Oxford). (2011) 50:1982–90. doi: 10.1093/rheumatology/ker017, PMID: 21393338

[3] Wang H Shui L Chen Y. Combine use of glucocorticoid with other immunosuppressants is a risk factor for pneumocystis jirovecii pneumonia in autoimmune inflammatory disease patients: a meta-analysis. Clin Rheumatol. (2023) 42:269–76. doi: 10.1007/s10067-022-06381-y, PMID: 36149536

[4] Park JW Curtis JR Moon J Song YW Kim S Lee EB. Prophylactic effect of trimethoprim-sulfamethoxazole for pneumocystis pneumonia in patients with rheumatic diseases exposed to prolonged high-dose glucocorticoids. Ann Rheum Dis. (2018) 77:644–9. doi: 10.1136/annrheumdis-2017-211796, PMID: 29092853 PMC5909751

[5] Minderhoud TC van Meer M van Thiel RJ den Hoed CM van Daele P Schurink C. Infections during glucocorticoid use. Ned Tijdschr Geneeskd. (2018) 162:D2215 30212002

[6] Agustí C Rañó A Filella X González J Moreno A Xaubet A. Pulmonary infiltrates in patients receiving long-term glucocorticoid treatment: etiology, prognostic factors, and associated inflammatory response. Chest. (2003) 123:488–98. doi: 10.1378/chest.123.2.488, PMID: 12576371

[7] Alfakeekh K Azar M Sowailmi BA Alsulaiman S Makdob SA Omair A. Immunosuppressive burden and risk factors of infection in primary childhood nephrotic syndrome. J Infect Public Health. (2019) 12:90–4. doi: 10.1016/j.jiph.2018.09.006, PMID: 30279098

[8] Park JW Curtis JR Kim MJ Lee H Song YW Lee EB. Pneumocystis pneumonia in patients with rheumatic diseases receiving prolonged, non-high-dose steroids-clinical implication of primary prophylaxis using trimethoprim-sulfamethoxazole. Arthritis Res Ther. (2019) 21:207. doi: 10.1186/s13075-019-1996-6, PMID: 31521185 PMC6744623

[9] Crim C Calverley PM Anderson JA Celli B Ferguson GT Jenkins C. Pneumonia risk in COPD patients receiving inhaled corticosteroids alone or in combination: TORCH study results. Eur Respir J. (2009) 34:641–7. doi: 10.1183/09031936.00193908 19443528

[10] Chew LC Maceda-Galang LM Tan YK Chakraborty B Thumboo J. Pneumocystis jirovecii pneumonia in patients with autoimmune disease on high-dose glucocorticoid. J Clin Rheumatol. (2015) 21:72–5. doi: 10.1097/RHU.0000000000000215, PMID: 25710857

[11] Liu Y Liu Y Dai J Liu A Li Y Xu J. *Klebsiella pneumoniae* pneumonia in patients with rheumatic autoimmune diseases: clinical characteristics, antimicrobial resistance and factors associated with extended-spectrum beta-lactamase production. BMC Infect Dis. (2021) 21:366. doi: 10.1186/s12879-021-06055-1, PMID: 33865323 PMC8053293

[12] Verhaert M Blockmans D De Langhe E Henckaerts L. Pneumocystis jirovecii pneumonia in patients treated for systemic autoimmune disorders: a retrospective analysis of patient characteristics and outcome. Scand J Rheumatol. (2020) 49:345–52. doi: 10.1080/03009742.2020.1762921, PMID: 32662308

[13] Westhoff PG De Ruysscher DK Schramel FM Bulbul M Dendooven A El SS. Fatal bilateral pneumonitis after locoregional thoracic chemoradiation in a transplanted patient under immunosuppressive therapy. Anticancer Res. (2014) 34:7315–7. PMID: 25503166

[14] Yang CY Yang AH Yang WC Lin CC. Risk factors for pneumocystis jiroveci pneumonia in glomerulonephritis patients receiving immunosuppressants. Intern Med. (2012) 51:2869–75. doi: 10.2169/internalmedicine.51.6774, PMID: 23064560

[15] Yoshida Y Takahashi Y Minemura N Ueda Y Yamashita H Kaneko H. Prognosis of pneumocystis pneumonia complicated in patients with rheumatoid arthritis (RA) and non-RA rheumatic diseases. Mod Rheumatol. (2012) 22:509–14. doi: 10.1007/s10165-011-0523-7, PMID: 21971942

[16] Falagas ME Manta KG Betsi GI Pappas G. Infection-related morbidity and mortality in patients with connective tissue diseases: a systematic review. Clin Rheumatol. (2007) 26:663–70. doi: 10.1007/s10067-006-0441-9, PMID: 17186117

[17] Ajmal S Mahmood M Abu SO Larson J Sohail MR. Invasive fungal infections associated with prior respiratory viral infections in immunocompromised hosts. Infection. (2018) 46:555–8. doi: 10.1007/s15010-018-1138-0, PMID: 29627936

[18] Korkmaz Ekren P Töreyin ZN Nahid P Doskaya M Caner A Turgay N. The association between Cytomegalovirus co-infection with Pneumocystis pneumonia and mortality in immunocompromised non-HIV patients. Clin Respir J. (2018) 12:2590–7. doi: 10.1111/crj.12961 30244544

[19] Asai N Motojima S Ohkuni Y Matsunuma R Iwasaki T Nakashima K. Clinical manifestations and prognostic factors of pneumocystis jirovecii pneumonia without HIV. Chemotherapy. (2017) 62:343–9. doi: 10.1159/000477332, PMID: 28719897

[20] Kotani T Katayama S Miyazaki Y Fukuda S Sato Y Ohsugi K. Risk factors for the mortality of pneumocystis jirovecii pneumonia in non-HIV patients who required mechanical ventilation: a retrospective case series study. Biomed Res Int. (2017) 2017:7452604. doi: 10.1155/2017/7452604, PMID: 28567422 PMC5439059

[21] Yu Q Jia P Su L Zhao H Que C. Outcomes and prognostic factors of non-HIV patients with pneumocystis jirovecii pneumonia and pulmonary CMV co-infection: a retrospective cohort study. BMC Infect Dis. (2017) 17:17. doi: 10.1186/s12879-017-2492-8, PMID: 28583135 PMC5460484

[22] Kim SJ Lee J Cho Y Park YS Lee C Yoon HI. Prognostic factors of pneumocystis jirovecii pneumonia in patients without HIV infection. J Infect. (2014) 69:88–95. doi: 10.1016/j.jinf.2014.02.015 24607411

[23] Kim T Moon SM Sung H Kim M Kim S Choi S. Outcomes of non-HIV-infected patients with pneumocystis pneumonia and concomitant pulmonary cytomegalovirus infection. Scand J Infect Dis. (2012) 44:670–7. doi: 10.3109/00365548.2011.652665, PMID: 22264016

[24] Rañó A Agustí C Benito N Rovira M Angrill J Pumarola T. Prognostic factors of non-HIV immunocompromised patients with pulmonary infiltrates. Chest. (2002) 122:253–61. doi: 10.1378/chest.122.1.253, PMID: 12114367

[25] Li L Hsu SH Gu X Jiang S Shang L Sun G. Aetiology and prognostic risk factors of mortality in pneumonia patients receiving glucocorticoids alone or glucocorticoids and other immunosuppressants: a retrospective cohort study, Dryad, Dataset. doi: 10.5061/dryad.mkkwh70x2, PMID: 33109645 PMC7592294

[26] Einarsdottir MJ Ekman P Molin M Trimpou P Olsson DS Johannsson G. High mortality rate in oral glucocorticoid users: a population-based matched cohort study. Front Endocrinol (Lausanne). (2022) 13:918356. doi: 10.3389/fendo.2022.918356, PMID: 35872995 PMC9304700

[27] Li L Hsu SH Gu X Jiang S Shang L Sun G. Aetiology and prognostic risk factors of mortality in patients with pneumonia receiving glucocorticoids alone or glucocorticoids and other immunosuppressants: a retrospective cohort study. BMJ Open. (2020) 10:e037419. doi: 10.5061/dryad.mkkwh70x2 PMC7592294 33109645

[28] Rano A Agusti C Sibila O Torres A. Pulmonary infections in non-HIV-immunocompromised patients. Curr Opin Pulm Med. (2005) 11:213–7. doi: 10.1097/01.mcp.0000158728.14945.46 15818182

[29] Roux A Gonzalez F Roux M Mehrad M Menotti J Zahar JR. Update on pulmonary pneumocystis jirovecii infection in non-HIV patients. Med Mal Infect. (2014) 44:185–98. doi: 10.1016/j.medmal.2014.01.007, PMID: 24630595

[30] Ewig S Bauer T Schneider C Pickenhain A Pizzulli L Loos U. Clinical characteristics and outcome of pneumocystis carinii pneumonia in HIV-infected and otherwise immunosuppressed patients. Eur Respir J. (1995) 8:1548–53. doi: 10.1183/09031936.95.08091548, PMID: 8575583

[31] Sundahl N Bridelance J Libert C De Bosscher K Beck IM. Selective glucocorticoid receptor modulation: new directions with non-steroidal scaffolds. Pharmacol Ther. (2015) 152:28–41. doi: 10.1016/j.pharmthera.2015.05.001, PMID: 25958032

[32] Matteson EL Buttgereit F Dejaco C Dasgupta B. Glucocorticoids for management of polymyalgia rheumatica and giant cell arteritis. Rheum Dis Clin N Am. (2016) 42:75–90. doi: 10.1016/j.rdc.2015.08.009 26611552

[33] Schacke H Docke WD Asadullah K. Mechanisms involved in the side effects of glucocorticoids. Pharmacol Ther. (2002) 96:23–43. doi: 10.1016/s0163-7258(02)00297-8 12441176

[34] Yale SH Limper AH. Pneumocystis carinii pneumonia in patients without acquired immunodeficiency syndrome: associated illness and prior corticosteroid therapy. Mayo Clin Proc. (1996) 71:5–13. doi: 10.4065/71.1.5, PMID: 8538233

[35] Maeda T Babazono A Nishi T Matsuda S Fushimi K Fujimori K. Quantification of the effect of chemotherapy and steroids on risk ofPneumocystis jiroveci among hospitalized patients with adult T-cell leukaemia. Br J Haematol. (2015) 168:501–6. doi: 10.1111/bjh.13154, PMID: 25266912

[36] Martin SI Fishman JA. Pneumocystis pneumonia in solid organ transplantation. Am J Transplant. (2013) 13:272–9. doi: 10.1111/ajt.12119 23465020

[37] Buttgereit F Matteson EL Dejaco C Dasgupta B. Prevention of glucocorticoid morbidity in giant cell arteritis. Rheumatology. (2018) 57:ii11–21. doi: 10.1093/rheumatology/kex459, PMID: 29982779

[38] Broder MS Sarsour K Chang E Collinson N Tuckwell K Napalkov P. Corticosteroid-related adverse events in patients with giant cell arteritis: a claims-based analysis. Semin Arthritis Rheum. (2016) 46:246–52. doi: 10.1016/j.semarthrit.2016.05.009, PMID: 27378247

[39] Del Rincón I Battafarano DF Restrepo JF Erikson JM Escalante A. Glucocorticoid dose thresholds associated with all-cause and cardiovascular mortality in rheumatoid arthritis. Arthritis Rheumatol. (2014) 66:264–72. doi: 10.1002/art.38210, PMID: 24504798

