# Supplementary material for: Association between the cumulative dose of glucocorticoids before the development of pneumonia and death in patients receiving long-term glucocorticoids: a secondary analysis based on a Chinese cohort study
Source: Front Med (Lausanne). 2023 Jul 20;10:1175855. doi: 10.3389/fmed.2023.1175855 (PMC10399627; doi:10.3389/fmed.2023.1175855)
Supplement: Supplementary file 1 [file Data_Sheet_1.doc]

**Supplementary:**

**Supplementary Table 1. Symptoms, signs, and laboratory tests according to the cumulative dose of GCs in study participants.**

**Supplementary Figure 1. Results of the subgroup analyses based on RCS.**

**Supplementary Table 2. Characteristics of participants after propensity-score matching.**

**Supplementary Table 3. Hazard ratios (95% CIs) for the association between the cumulative dose of GCs and 30-day death by COX regression models after propensity-score matching.**

**Supplementary Figure 2. The smoothing curve of the dose-response relationship between the cumulative dose of GCs and 30-day death after propensity-score matching.**

**Supplementary Table 4. Hazard ratios (95% CIs) for the association between the cumulative dose of GCs and 90-day death by COX regression models.**

**Supplementary Figure 3. The smoothing curve of the dose-response relationship between the cumulative dose of GCs and 90-day death.**

**Supplementary Table 1. Symptoms, signs, and laboratory tests according to the cumulative dose of GCs in study participants**

| **Variables** | **Total(N=625)** | **Q1(N=125)** | **Q2(N=130)** | **Q3(N=120)** | **Q4(N=125)** | **Q5(N=125)** | ***P* value** |
| --- | --- | --- | --- | --- | --- | --- | --- |
| **Symptoms and signs, n (%)** |  |  |  |  |  |  |  |
| Fever | 472 (75.5) | 94 (75.2) | 99 (76.2) | 100 (83.3) | 85 (68.0) | 94 (75.2) | 0.098 |
| Cough | 551 (88.2) | 117 (93.6) | 111 (85.4) | 110 (91.7) | 108 (86.4) | 105 (84.0) | 0.079 |
| Sputum production | 512 (82.1) | 112 (89.6) | 104 (80.6) | 103 (85.8) | 95 (76.0) | 98 (78.4) | 0.034 |
| Dyspnoea | 392 (62.7) | 84 (67.2) | 85 (65.4) | 83 (69.2) | 75 (60.0) | 65 (52.0) | 0.038 |
| Disturbance of consciousness | 32 ( 5.1) | 6 ( 4.8) | 7 ( 5.4) | 9 ( 7.5) | 4 ( 3.2) | 6 ( 4.8) | 0.659 |
| **Laboratory examination** |  |  |  |  |  |  |  |
| White cell, ×10^9^/L (median[IQR]) | 7.98 [5.73, 11.53] | 8.54 [5.71, 13.19] | 7.74 [5.70, 11.16] | 9.27 [6.88, 11.73] | 7.73 [6.04, 11.43] | 7.14 [5.01, 9.23] | 0.003 |
| Neutrophils, ×10^9^/L (median[IQR]) | 6.61 [4.33, 10.08] | 7.03 [4.40, 10.94] | 6.30 [4.43, 9.25] | 7.53 [5.27, 10.47] | 6.85 [4.28, 10.00] | 5.69 [3.46, 7.87] | 0.003 |
| Lymphocyte, ×10^9^/L (median[IQR]) | 0.83 [0.49, 1.37] | 0.78 [0.40, 1.31] | 0.78 [0.50, 1.34] | 0.86 [0.50, 1.39] | 0.91 [0.57, 1.33] | 0.83 [0.45, 1.40] | 0.836 |
| Haemoglobin,g/L (mean±SD) | 111.48 (23.64) | 107.78 (23.40) | 113.01 (24.74) | 115.27 (23.36) | 113.20 (24.98) | 108.14 (20.89) | 0.045 |
| Albumin, g/L (mean±SD) | 33.01 (6.31) | 33.73 (7.10) | 32.15 (6.64) | 31.79 (6.32) | 33.82 (5.61) | 33.61 (5.53) | 0.028 |
| Lactate dehydrogenase,U/L (median[IQR]) | 345.00 [235.00, 506.00] | 427.00 [257.00, 556.00] | 387.00 [288.00, 506.50] | 395.00 [266.50, 574.50] | 292.00 [225.00, 475.00] | 262.00 [192.50, 338.75] | <0.001 |
| Blood urea nitrogen, mmol/L (median[IQR]) | 6.29 [4.65, 10.30] | 7.78 [5.26, 12.40] | 7.36 [5.20, 11.58] | 6.25 [5.08, 9.01] | 5.40 [4.46, 7.76] | 5.43 [4.05, 9.08] | <0.001 |
| Serum creatinine, mmol/L (median[IQR]) | 64.30 [50.38, 93.53] | 62.70 [50.20, 95.80] | 69.70 [55.40, 100.20] | 57.60 [46.95, 82.30] | 62.40 [49.95, 88.83] | 67.00 [53.30, 111.80] | 0.046 |
| Procalcitonin, ng/mL (median[IQR]) | 0.28 [0.12, 0.77] | 0.31 [0.13, 0.70] | 0.29 [0.11, 0.95] | 0.27 [0.11, 0.74] | 0.26 [0.12, 0.62] | 0.32 [0.15, 0.79] | 0.79 |
| Oxygenation index (median[IQR]) | 234.39 [123.82, 346.34] | 221.19 [116.73, 310.26] | 211.33 [110.83, 331.67] | 190.25 [120.00, 342.86] | 248.10 [138.33, 367.86] | 280.95 [183.33, 361.63] | 0.032 |


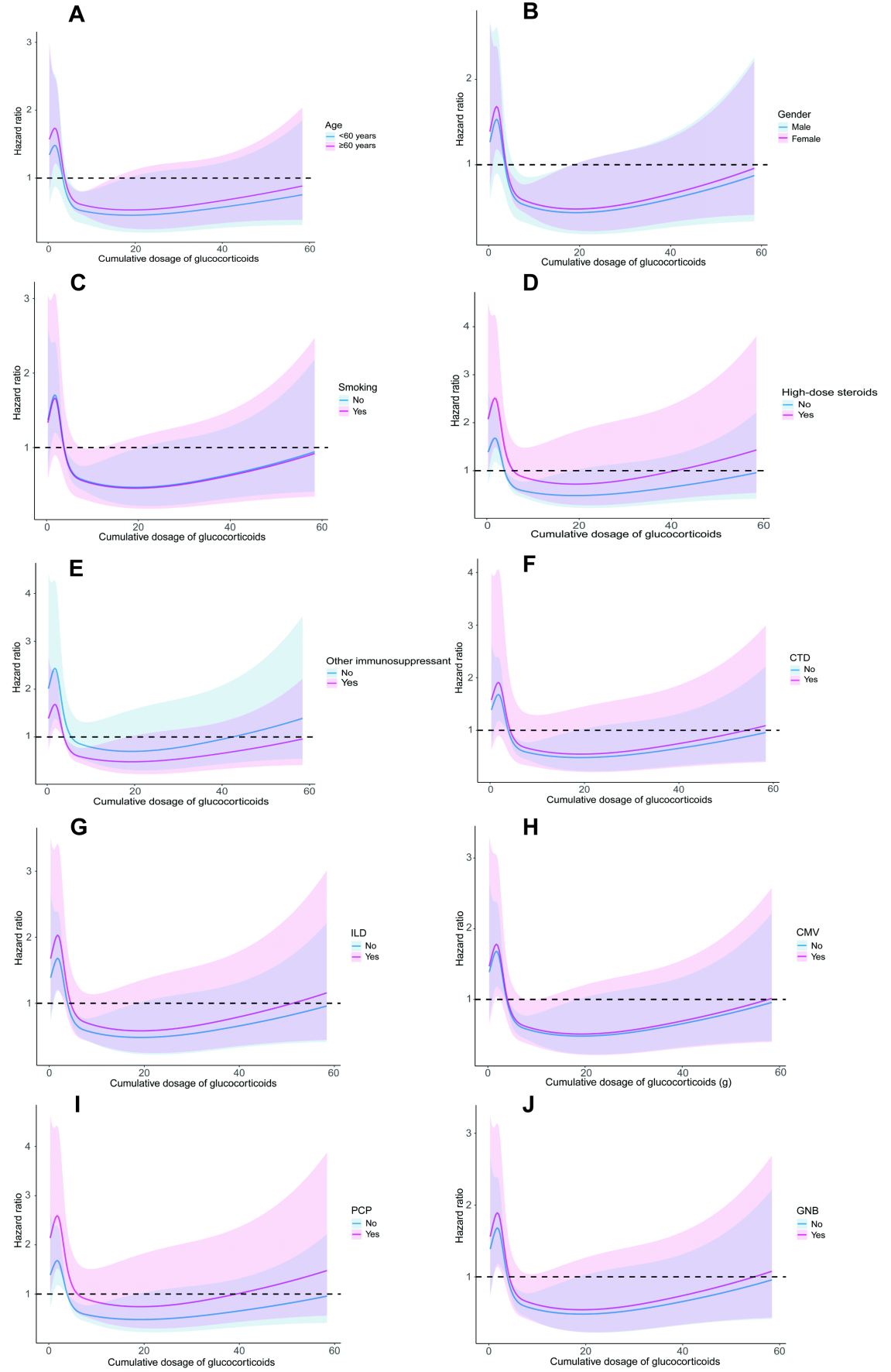


**Supplementary Figure 1. Results of** **the subgroup analyses based on RCS.** Adjusted for age, sex, smoking status, drinking status, high-dose steroids use, receiving other immunosuppressants, Pre-admission antibiotics, Pre-admission antiviral drugs, Diabetes mellitus, Tumor, Connective tissue disease, Interstitial lung disease, Nephrotic syndrome or chronic glomerulonephritis, Idiopathic interstitial pneumonia, Bronchial asthma or chronic obstructive pulmonary disease, Lymphoma, Bone marrow or hematopoietic stem cell transplant, Solid organ transplant, Radiation pneumonitis, Microbial etiology of pneumonia (including cytomegalovirus, influenza virus, respiratory syncytial virus, pneumocystis, aspergillus, Gram-negative and Gram-positive bacteria). CTD, Connective tissue disease; ILD, Interstitial lung disease; CMV, Cytomegalovirus; PCP, Pneumocystis pneumonia; GNB, Gram-negative bacteria.

**Supplementary Table 2. Characteristics of participants after propensity-score matching**

| **Variables, n (%)** | **Total**  **(N=315)** | **Non-death**  **(N=196)** | **Death**  **(N=119)** | ***P* value** |
| --- | --- | --- | --- | --- |
| **Age** |  |  |  | 0.956 |
| 18-39 | 22 ( 7.0) | 12 ( 6.1) | 10 ( 8.4) |  |
| 40-49 | 39 (12.4) | 25 (12.8) | 14 (11.8) |  |
| 50-59 | 72 (22.9) | 47 (24.0) | 25 (21.0) |  |
| 60-69 | 106 (33.7) | 64 (32.7) | 42 (35.3) |  |
| 70-79 | 49 (15.6) | 31 (15.8) | 18 (15.1) |  |
| 80-99 | 27 ( 8.6) | 17 ( 8.7) | 10 ( 8.4) |  |
| **Sex, Female** | 139 (44.1) | 85 (43.4) | 54 (45.4) | 0.817 |
| **Smoking** |  |  |  | 0.948 |
| Never | 224 (71.1) | 139 (70.9) | 85 (71.4) |  |
| Current | 84 (26.7) | 53 (27.0) | 31 (26.1) |  |
| Former | 7 ( 2.2) | 4 ( 2.0) | 3 ( 2.5) |  |
| Alcoholism | 27 ( 8.6) | 17 ( 8.7) | 10 ( 8.4) | 1 |
| High-dose steroids | 137 (43.5) | 83 (42.3) | 54 (45.4) | 0.683 |
| Other immunosuppressant | 119 (37.8) | 78 (39.8) | 41 (34.5) | 0.408 |
| Pre-admission antibiotics | 228 (72.4) | 139 (70.9) | 89 (74.8) | 0.538 |
| Pre-admission antiviral drugs | 47 (14.9) | 25 (12.8) | 22 (18.5) | 0.222 |
| Diabetes mellitus | 81 (25.7) | 48 (24.5) | 33 (27.7) | 0.613 |
| Tumor | 22 ( 7.0) | 13 ( 6.6) | 9 ( 7.6) | 0.931 |
| Connective tissue disease | 183 (58.1) | 112 (57.1) | 71 (59.7) | 0.748 |
| Interstitial lung disease | 166 (52.7) | 102 (52.0) | 64 (53.8) | 0.854 |
| Nephrotic syndrome or chronic glomerulonephritis | 42 (13.3) | 28 (14.3) | 14 (11.8) | 0.64 |
| Idiopathic interstitial pneumonia | 30 ( 9.5) | 18 ( 9.2) | 12 (10.1) | 0.947 |
| Bronchial asthma or chronic obstructive pulmonary disease | 6 ( 1.9) | 4 ( 2.0) | 2 ( 1.7) | 1 |
| Lymphoma | 6 ( 1.9) | 5 ( 2.6) | 1 ( 0.8) | 0.515 |
| Bone marrow or hematopoietic stem cell transplant, | 2 ( 0.6) | 1 ( 0.5) | 1 ( 0.8) | 1 |
| Solid organ transplant | 15 ( 4.8) | 8 ( 4.1) | 7 ( 5.9) | 0.649 |
| Radiation pneumonitis | 4 ( 1.3) | 3 ( 1.5) | 1 ( 0.8) | 0.991 |
| **Pathogens** |  |  |  |  |
| **Virus** |  |  |  |  |
| Cytomegalovirus | 116 (36.8) | 73 (37.2) | 43 (36.1) | 0.938 |
| Influenza virus | 29 ( 9.2) | 15 ( 7.7) | 14 (11.8) | 0.306 |
| Respiratory syncytial virus | 23 ( 7.3) | 15 ( 7.7) | 8 ( 6.7) | 0.933 |
| **Fungus** |  |  |  |  |
| Pneumocystis | 89 (28.3) | 57 (29.1) | 32 (26.9) | 0.772 |
| Aspergillus | 45 (14.3) | 25 (12.8) | 20 (16.8) | 0.406 |
| **Bacteriums** |  |  |  |  |
| Gram-negative | 99 (31.4) | 60 (30.6) | 39 (32.8) | 0.783 |
| Gram-positive | 25 ( 7.9) | 14 ( 7.1) | 11 ( 9.2) | 0.65 |

**Supplementary Table 3. Hazard ratios (95% CIs) for the association between the cumulative dose of GCs and 30-day death by COX regression models** **after propensity-score matching**

|  | **Unadjusted** | |  | **Adjusted*** | |
| --- | --- | --- | --- | --- | --- |
|  | **HR (95% CI)** | ***P* value** |  | **HR (95% CI)** | ***P* value** |
| Q1 | 1 | - |  | 1 | - |
| Q2 | 0.95 (0.58, 1.56) | 0.834 |  | 0.93 (0.56, 1.53) | 0.766 |
| Q3 | 0.81 (0.49, 1.34) | 0.411 |  | 0.80 (0.48, 1.33) | 0.387 |
| Q4 | 0.28 (0.14, 0.57) | <0.001 |  | 0.29 (0.14, 0.57) | <0.001 |
| Q5 | 0.50 (0.27, 0.91) | 0.025 |  | 0.50 (0.27, 0.92) | 0.026 |

*Adjusted for the propensity score.


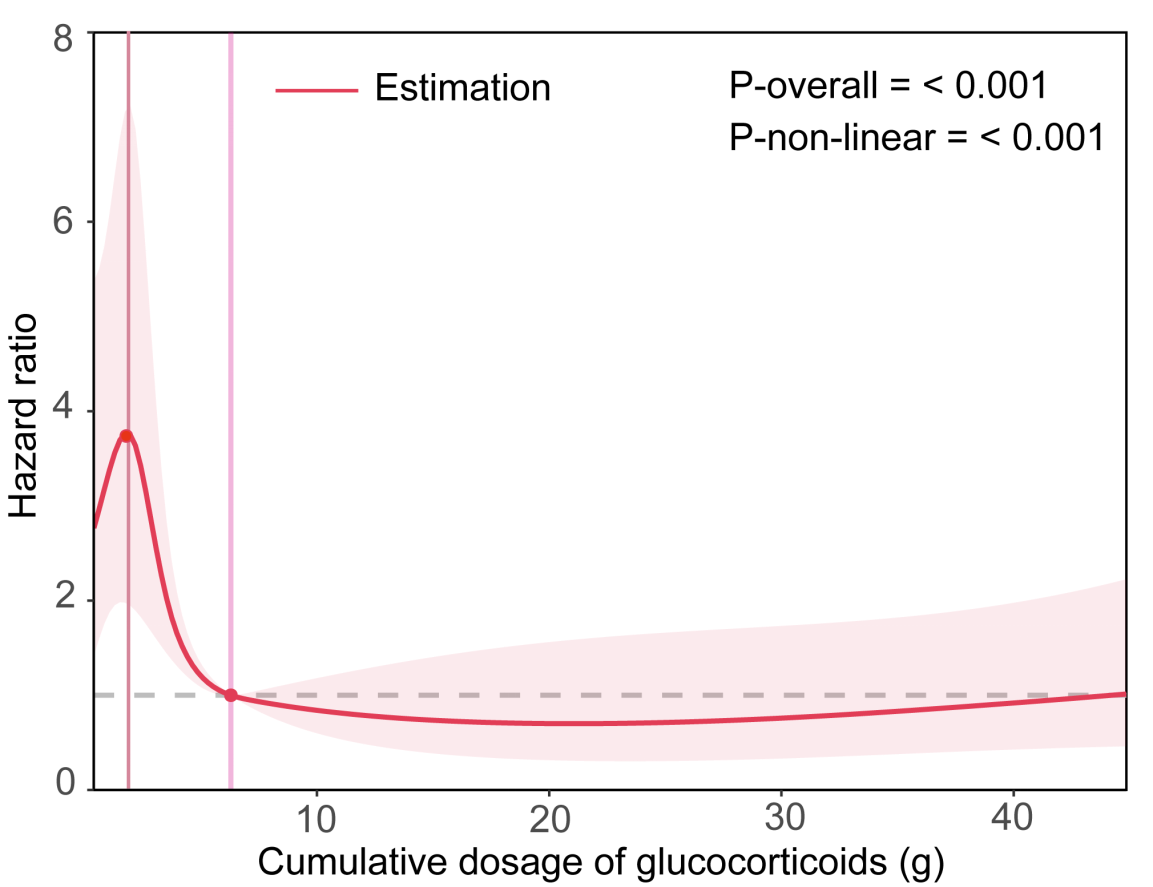


**Supplementary Figure 2. The smoothing curve of the dose-response relationship between the cumulative dose of GCs and 30-day death after propensity-score matching.** Adjusted for age, sex, smoking status, drinking status, high-dose steroids use, receiving other immunosuppressants, Pre-admission antibiotics, Pre-admission antiviral drugs, Diabetes mellitus, Tumor, Connective tissue disease, Interstitial lung disease, Nephrotic syndrome or chronic glomerulonephritis, Idiopathic interstitial pneumonia, Bronchial asthma or chronic obstructive pulmonary disease, Lymphoma, Bone marrow or hematopoietic stem cell transplant, Solid organ transplant, Radiation pneumonitis, Microbial etiology of pneumonia (including cytomegalovirus, influenza virus, respiratory syncytial virus, pneumocystis, aspergillus, Gram-negative and Gram-positive bacteria). The solid line represents the HRs, and the shaded areas represent the 95% confidence intervals for the spline model.

**Supplementary Table 4. Hazard ratios (95% CIs) for the association between the cumulative dose of GCs and 90-day death by COX regression models**

|  | **Crude** | |  | **Adjust Model Ⅰ*** | |  | **Adjust Model Ⅱ**** | |
| --- | --- | --- | --- | --- | --- | --- | --- | --- |
|  | **HR (95% CI)** | ***P* value** |  | **HR (95% CI)** | ***P* value** |  | **HR (95% CI)** | ***P* value** |
| Q1 | 1 | - |  | 1 | - |  | 1 | - |
| Q2 | 0.96 (0.62, 1.48) | 0.851 |  | 0.97 (0.63, 1.50) | 0.894 |  | 0.88 (0.55, 1.42) | 0.613 |
| Q3 | 1.03 (0.67, 1.59) | 0.877 |  | 1.06 (0.68, 1.63) | 0.805 |  | 0.84 (0.53, 1.34) | 0.475 |
| Q4 | 0.47 (0.28, 0.79) | 0.004 |  | 0.47 (0.28, 0.79) | 0.004 |  | 0.38 (0.22, 0.67) | 0.001 |
| Q5 | 0.39 (0.22, 0.67) | 0.001 |  | 0.39 (0.22, 0.68) | 0.001 |  | 0.38 (0.20, 0.69) | 0.002 |

*.Adjusted for age, sex, smoking, Alcoholism.

**Adjusted for age, sex, smoking status, drinking status, high-dose steroids use, receiving other immunosuppressants, Pre-admission antibiotics, Pre-admission antiviral drugs, Diabetes mellitus, Tumor, Connective tissue disease, Interstitial lung disease, Nephrotic syndrome or chronic glomerulonephritis, Idiopathic interstitial pneumonia, Bronchial asthma or chronic obstructive pulmonary disease, Lymphoma, Bone marrow or hematopoietic stem cell transplant, Solid organ transplant, Radiation pneumonitis, Microbial etiology of pneumonia (including cytomegalovirus, influenza virus, respiratory syncytial virus, pneumocystis, aspergillus, Gram-negative and Gram-positive bacteria). Q, quintile.


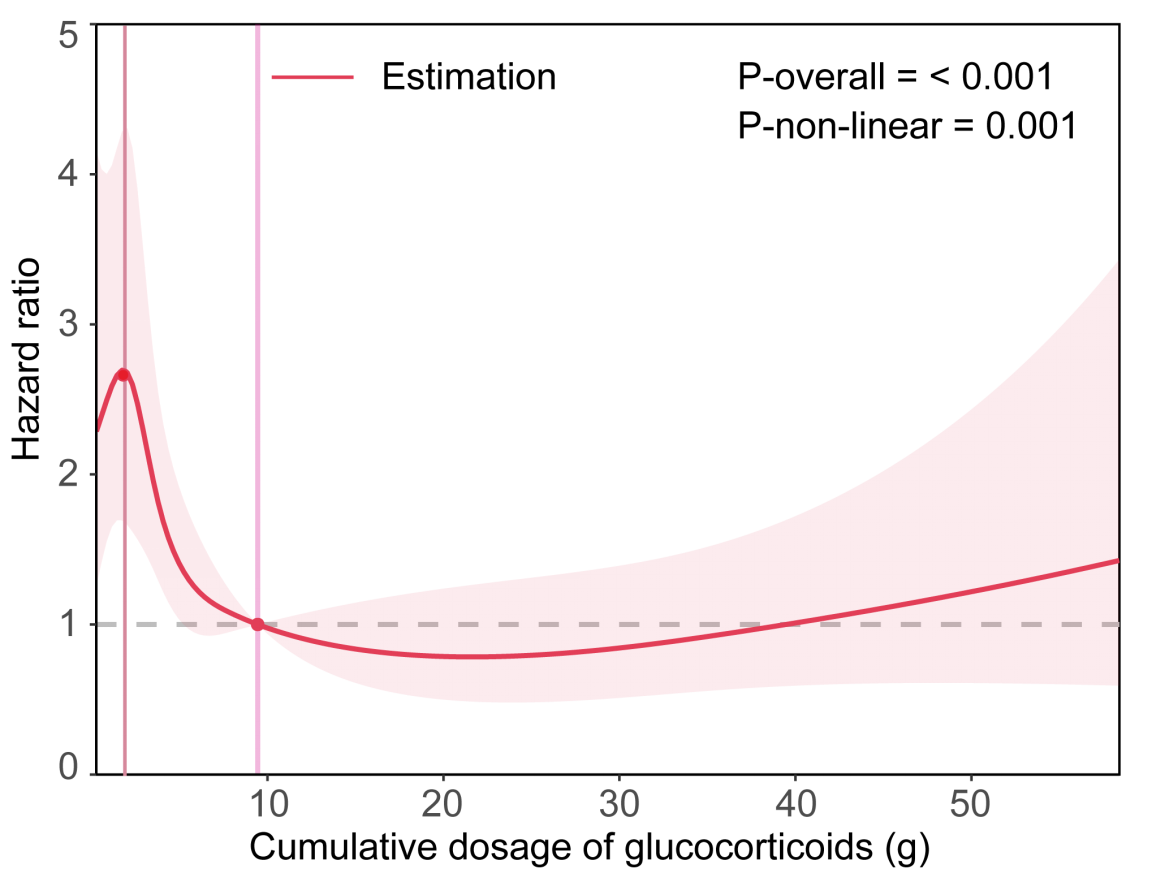


**Supplementary Figure 3. The smoothing curve of the dose-response relationship between the cumulative dose of GCs and 90-day death.** Adjusted for age, sex, smoking status, drinking status, high-dose steroids use, receiving other immunosuppressants, Pre-admission antibiotics, Pre-admission antiviral drugs, Diabetes mellitus, Tumor, Connective tissue disease, Interstitial lung disease, Nephrotic syndrome or chronic glomerulonephritis, Idiopathic interstitial pneumonia, Bronchial asthma or chronic obstructive pulmonary disease, Lymphoma, Bone marrow or hematopoietic stem cell transplant, Solid organ transplant, Radiation pneumonitis, Microbial etiology of pneumonia (including cytomegalovirus, influenza virus, respiratory syncytial virus, pneumocystis, aspergillus, Gram-negative and Gram-positive bacteria). The solid line represents the HRs, and the shaded areas represent the 95% confidence intervals for the spline model.
